# Circular RNA mitochondrial translation optimization 1 correlates with less lymph node metastasis, longer disease‐free survival, and higher chemotherapy sensitivity in gastric cancer

**DOI:** 10.1002/jcla.23918

**Published:** 2022-04-27

**Authors:** Cheng Chang, Anrui Zheng, Pinfa Wang, Xiaojun Teng

**Affiliations:** ^1^ Department of Gastroenterology Wuhan Hospital of Traditional Chinese medicine Wuhan China; ^2^ Department of Gastroenterology Huangshi Central Hospital, Affiliated Hospital of Hubei Polytechnic University, Edong Healthcare Group Huangshi China

**Keywords:** Circ‐MTO1, DFS, Gastric cancer, Oxaliplatin sensitivity, TNM stage

## Abstract

**Objective:**

Circular‐mitochondrial translation optimization 1 (circ‐MTO1) inhibits the progression of gastric cancer by regulating the growth, apoptosis, and invasion of tumor cells. However, its clinical potential as a biomarker for gastric cancer remains to be further evaluated. This study aimed to assess circ‐MTO1 expression and its correlation with clinical features and prognosis in gastric cancer patients, as well as the effect of circ‐MTO1 on the sensitivity to chemotherapy in gastric cancer cells.

**Methods:**

Circ‐MTO1 in tumor and adjacent tissues of 97 gastric cancer patients undergoing resection was examined by reverse transcription‐quantitative polymerase chain reaction. HGC‐27 and NCI‐N87 cells transfected by circ‐MOT1 overexpression plasmid (OE‐circ‐MOT1) and negative control (OE‐NC) were treated with 0‒6.4 μM oxaliplatin. Relative cell viability was detected using Cell Counting Kit‐8.

**Results:**

Circ‐MTO1 was insufficiently expressed in gastric tumor tissue (median (interquartile range): 0.403 (0.288‒0.518)) compared with adjacent tissue (median (interquartile range): 1.000 (0.715‒1.524)) (*p* < 0.001). Besides, tumor circ‐MTO1 was correlated with less lymph node metastasis (*p* = 0.014) and low TNM stage (*p* = 0.039), while was not correlated with demographic features or other clinical characteristics (all *p* > 0.05). Furthermore, tumor circ‐MTO1 high expression was independently correlated with prolonged disease‐free survival (DFS) (*p* = 0.013, adjusted hazard ratio (95% confidential interval): 0.314 (0.126‒0.782)), but was not correlated with overall survival (*p* > 0.05). Lastly, in gastric cancer cells, OE‐circ‐MTO1 apparently decreased relative cell viabilities at oxaliplatin concentrations of 0.4, 0.8, 1.6, and 3.2 μM (all *p* < 0.05).

**Conclusion:**

Circ‐MTO1 correlates with less lymph node metastasis, prolonged DFS, and improved chemotherapy sensitivity in gastric cancer.

## Graphical abstract

Circular‐mitochondrial translation optimization 1 (circ‐MTO1) inhibits the progression of gastric cancer by regulating the growth, apoptosis, and invasion of tumor cells. However, its clinical potential as a biomarker for gastric cancer remains to be further evaluated. This study aimed to assess circ‐MTO1 expression and its correlation with clinical features and prognosis in gastric cancer patients, as well as the effect of circ‐MTO1 on the sensitivity to chemotherapy in gastric cancer cells. Circ‐MTO1 in tumor and adjacent tissues of 97 gastric cancer patients undergoing resection were examined by reverse transcription‐quantitative polymerase chain reaction. HGC‐27 and NCI‐N87 cells transfected by circ‐MOT1 overexpression plasmid (OE‐circ‐MOT1) and negative control (OE‐NC) were treated with 0‒6.4 μM oxaliplatin. Relative cell viability was detected using Cell Counting Kit‐8. Circ‐MTO1 was insufficiently expressed in gastric tumor tissue (median (interquartile range): 0.403 (0.288‒0.518)) compared with adjacent tissue (median (interquartile range): 1.000 (0.715‒1.524)) (*p *< 0.001). Besides, tumor circ‐MTO1 was correlated with less lymph node metastasis (*p *= 0.014) and low TNM stage (*p *= 0.039), while was not correlated with demographic features or other clinical characteristics (all *p *> 0.05). Furthermore, tumor circ‐MTO1 high expression was independently correlated with prolonged disease‐free survival (DFS) (*p *= 0.013, adjusted hazard ratio (95% confidential interval): 0.314 (0.126‒0.782)), but was not correlated with overall survival (*p *> 0.05). Lastly, in gastric cancer cells, OE‐circ‐MTO1 apparently decreased relative cell viabilities at oxaliplatin concentrations of 0.4, 0.8, 1.6, and 3.2 μM (all *P*<0.05). Circ‐MTO1 correlates with less lymph node metastasis, prolonged DFS, and improved chemotherapy sensitivity in gastric cancer.

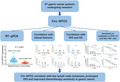

## 1 INTRODUCTION

Gastric cancer, originating from gastric mucosa epithelium, is a worldwide health burden since it is one of the top leading causes of cancer‐related mortality in humans^1^, ^2^ with over 950,000 newly diagnosed cases around the world annually (approximately 60% of new cases in east Asia such as China, Japan, and Korea).^3^ Over the past decades, progresses on the screening and diagnosis of gastric cancer bring an increased number of early‐stage gastric cancer patients.^4^, ^5^ These early‐stage patients, together with intermediate patients with gastric cancer, are recommended to be treated with gastric resection.^6^, ^7^, ^8^ Besides, neoadjuvant or adjuvant treatments such as chemoradiotherapy are supposed to be applied to creating conditions for surgical resection.^9^, ^10^ In addition, a huge number of studies are devoted to making contributions to the treatment for advanced gastric patients including chemotherapy, targeted therapy, and immunotherapy.^11^, ^12^, ^13^, ^14^ In spite of these, patients still commonly experience dissatisfied survivals.^15^ Therefore, finding biomarkers for monitoring the progression and prognosis of gastric cancer is important for more accurate treatment and better prognosis.

Circular RNAs (CircRNAs) are evolutionarily conserved molecules with covalently closed continuous loop structures that possess potential bioactivities in various cancers.^16^, ^17^, ^18^ For example, circRNAs modulate the proliferation, progression, tumorigenesis, and chemosensitivity of pancreatic cancer.^16^ Besides, circ_0016760 regulates the microRNA_1287/G antigen 1 signaling axis in lung cancer.^17^ Circular RNA mitochondrial translation optimization 1 (Circ‐MTO1) has been shown to be an anti‐tumor gene in cancers including gastric, colorectal, hepatocellular, and prostate cancers.^19^, ^20^, ^21^, ^22^, ^23^, ^24^ As for gastric cancer, previous studies exhibit that circ‐MTO1 suppresses its development and progression by regulating microRNA (miR)‐3200‐5p/phosphatidylethanolamine binding protein 1 (PEBP1) axis and miR‐199a‐3p/pro‐apoptotic WT1 regulator (PAWR) axis.^22^, ^23^ Clinically, insufficient circ‐MTO1 expression is correlated with frequent lymph node metastasis, advanced TNM stage, and shortened overall survival (OS) in patients with colorectal cancer.^19^ Besides, circ‐MTO1 high expression is correlated with decreased pathological T/N stage, as well as longer disease‐free survival (DFS) and OS in prostate cancer patients.^20^ However, the clinical potential of circ‐MTO1 as a biomarker for gastric cancer patients remains to be further evaluated.

This study aimed to assess the expression of circ‐MTO1, its correlation with clinical characteristics, and prognosis in postoperative patients with gastric cancer, as well as the effect of circ‐MTO1 on oxaliplatin sensitivity in gastric cancer cell lines.

## 2 MATERIALS AND METHODS

### 2.1 Patients

Ninety‐seven patients with gastric cancer who underwent surgical resection in our hospital from January 2016 to December 2019 were retrospectively included in this study. All patients were screened out from the electronic medical record system (EMRS), according to the following criteria: (i) pathologically confirmed as gastric cancer; (ii) received surgical resection without neoadjuvant therapy; (iii) had freshly frozen specimens including tumor and adjacent tissue; (iv) had complete clinicopathological information and follow‐up records; and (v) without other primary cancers or malignant diseases. Approval was obtained from the Ethical Inspection Committee, and the informed consent was signed by patients or their relatives.

### 2.2 Data acquisition

Clinical features were collected from the EMRS, including demographics, smoke and drink status, complications, helicobacter pylori infection status, tumor location, pathological grade, tumor size, clinical T stage, clinical N stage, clinical TNM stage, and adjuvant treatment (chemotherapy with XELOX regimen or S‐1 monotherapy, with or without radiotherapy). In addition, follow‐up information of patients was also reviewed to abstract the main time points for estimation of progression‐free survival (PFS) and overall survival (OS).

### 2.3 Circ‐MTO1 determination

The freshly frozen tumor and adjacent tissues of patients were acquired from the sample library of the hospital, which were available for reverse transcriptase quantitative polymerase chain reaction (RT‐qPCR). Briefly, total RNA was extracted from tumor or adjacent tissues by TRIzol™ Reagent (Thermo Fisher Scientific), followed by the removal of linear RNA with RNase R (Epicentre), and reverse transcription into cDNA using PrimeScript™ RT reagent Kit (Perfect Real Time) (Takara). Subsequently, qPCR was conducted by TB Green™ Fast qPCR Mix (Takara) to quantify the circ‐MTO1 expressions, which was calculated by 2^−ΔΔ^ *^C^* ^t^ method with glyceraldehyde‐3‐phosphate dehydrogenase (GAPDH) as an internal reference. Primers for PCR were designed according to previous studies.^22^, ^23^

### 2.4 Cell culture

The gastric cancer cell lines including HGC‐27 and NCI‐N87 were purchased from Cell Blank of Chinese Academy of Sciences (Shanghai). The cells were all cultured in the 90% RPMI1640 medium (Gibco) with 10% Fetal Bovine Serum (Gibco) and were maintained in at 37℃ in a humidified atmosphere of 5% CO_2_.

### 2.5 Plasmid transfection, treatment, and cell viability determination

The pCD5‐ciR vector (GENESEED) was applied to construct the circ‐MTO1 overexpression (OE) plasmid (OE‐circ‐MTO1) and negative control (OE‐NC) plasmid; then, the constructed plasmids were transfected into the HGC‐27 and NCI‐N87 cells using Lipofectamine™ 3000 Transfection Reagent (Thermo Fisher Scientific). The resulting cells were termed as OE‐circ‐MTO1 and OE‐NC, respectively. Meanwhile, the cells without transfection were used as blank controls. After transfection, all cells including OE‐circ‐MTO1, OE‐NC, and blank cells were treated with oxaliplatin (Sigma‐Aldrich) at the following concentrations: 0, 0.2, 0.4, 0.8, 1.6, 3.2, and 6.4 μM. After incubation for 48 h, the relative cell viability in each cell group was detected using Cell Counting Kit‐8 (Beyotime) as per the instruction of the kit.

### 2.6 Statistical analysis

Data were analyzed by SPSS 26.0 (IBM Corp.), and figures were plotted by GraphPad Prism 7.01 (GraphPad Software Inc.). Comparison analysis was determined by t test, Wilcoxon signed rank test, Wilcoxon rank sum test, or Kruskal‐Wallis H rank sum test. Correlation analysis was checked by Spearman rank correlation test. PFS and OS were displayed using Kaplan‐Meier method and determined by log‐rank test. Multivariable Cox's proportional hazard regression model analysis was applied to estimate the prognostic role of variables. *p* Value <0.05 was used as threshold to identify the statistical significance.

## 3 RESULTS

### 3.1 Clinical characteristics

The mean age of gastric cancer patients was 60.5 ± 11.5 years, among which female and male proportions were 45.4% and 54.6%, respectively. Regarding pathological grade, the number of gastric cancer patients with grade 1, grade 2, and grade 3 was 11 (11.3%), 72 (74.2%), and 14 (14.4%), respectively. With respect to clinical TNM stage, the number of gastric cancer patients with TNM stage I, II, and III was 20 (20.6%), 35 (36.1%), and 42 (43.3%), respectively. More detailed demographic and clinical characteristics were presented in Table 1.

**TABLE 1:** Clinical characteristics

Items	Gastric carcinoma patients (N = 97)
Age (years), mean±SD	60.5±11.5
Gender, No. (%)	
Female	44 (45.4)
Male	53 (54.6)
Current smoke, No. (%)	27 (27.8)
Current drink, No. (%)	37 (38.1)
Complications	
Hypertension, No. (%)	25 (25.8)
Hyperlipidemia, No. (%)	26 (26.8)
DM, No. (%)	14 (14.4)
Helicobacter pylori infection, No. (%)	
Negative	64 (66.0)
Positive	33 (34.0)
Tumor location, No. (%)	
Cardia	26 (26.8)
Gastric body	12 (12.4)
Gastric antrum	59 (60.8)
Pathological grade, No. (%)	
Grade 1	11 (11.3)
Grade 2	72 (74.2)
Grade 3	14 (14.4)
Tumor size (cm), median (IQR)	3.0 (2.5–4.0)
Clinical T stage, No. (%)	
T1	7 (7.2)
T2	18 (18.6)
T3	71 (73.2)
T4	1 (1.0)
Clinical N stage, No. (%)	
N0	50 (51.5)
N1	25 (25.8)
N2	18 (18.6)
N3	4 (4.1)
Clinical TNM stage, No. (%)	
Stage I	20 (20.6)
Stage II	35 (36.1)
Stage III	42 (43.3)
Adjuvant treatment	
Chemotherapy, No. (%)	74 (76.3)
Radiotherapy, No. (%)	9 (9.3)

Abbreviations: DM, diabetes mellitus;IQR, interquartile range; SD, standard deviation.

### 3.2 Circ‐MTO1 expression in tumor tissue and adjacent tissue

**FIGURE 1:**
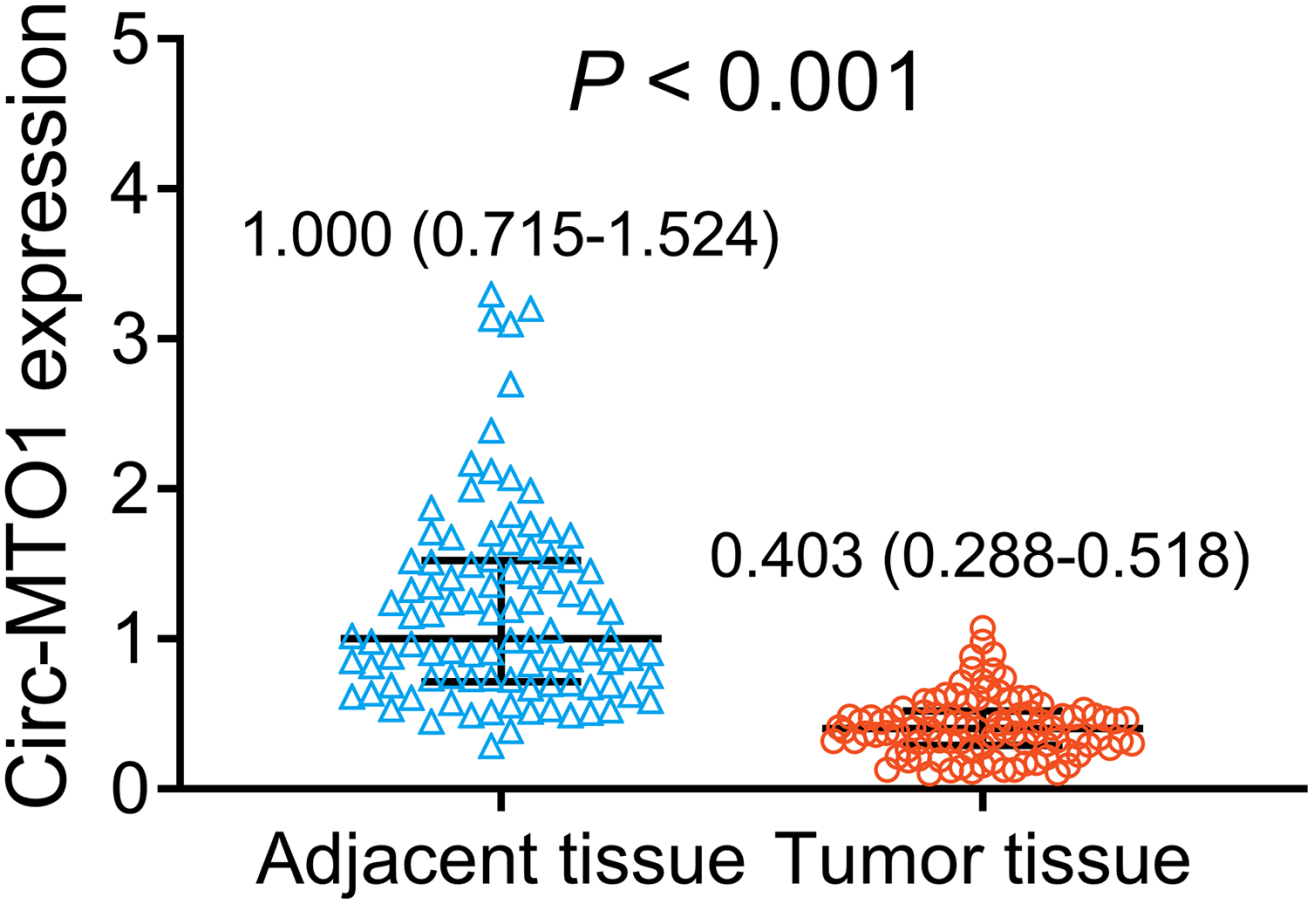
Circ‐MTO1 expression in gastric cancer patients. Comparison of circ‐MTO1 expression in adjacent tissue and tumor tissue of gastric cancer patients. Circ‐MTO1, circular RNA mitochondrial translation optimization 1

Circ‐MTO1 expression was obviously lower in tumor tissue than in adjacent tissue of gastric cancer patients (*p *< 0.001). The median (IQR) value of circ‐MTO1 expression in tumor tissue was 0.403 (0.288–0.518), while that in adjacent tissue was 1.000 (0.715–1.524) (Figure 1).

### 3.3 Correlation of tumor circ‐MTO1 with clinical features

**FIGURE 2:**
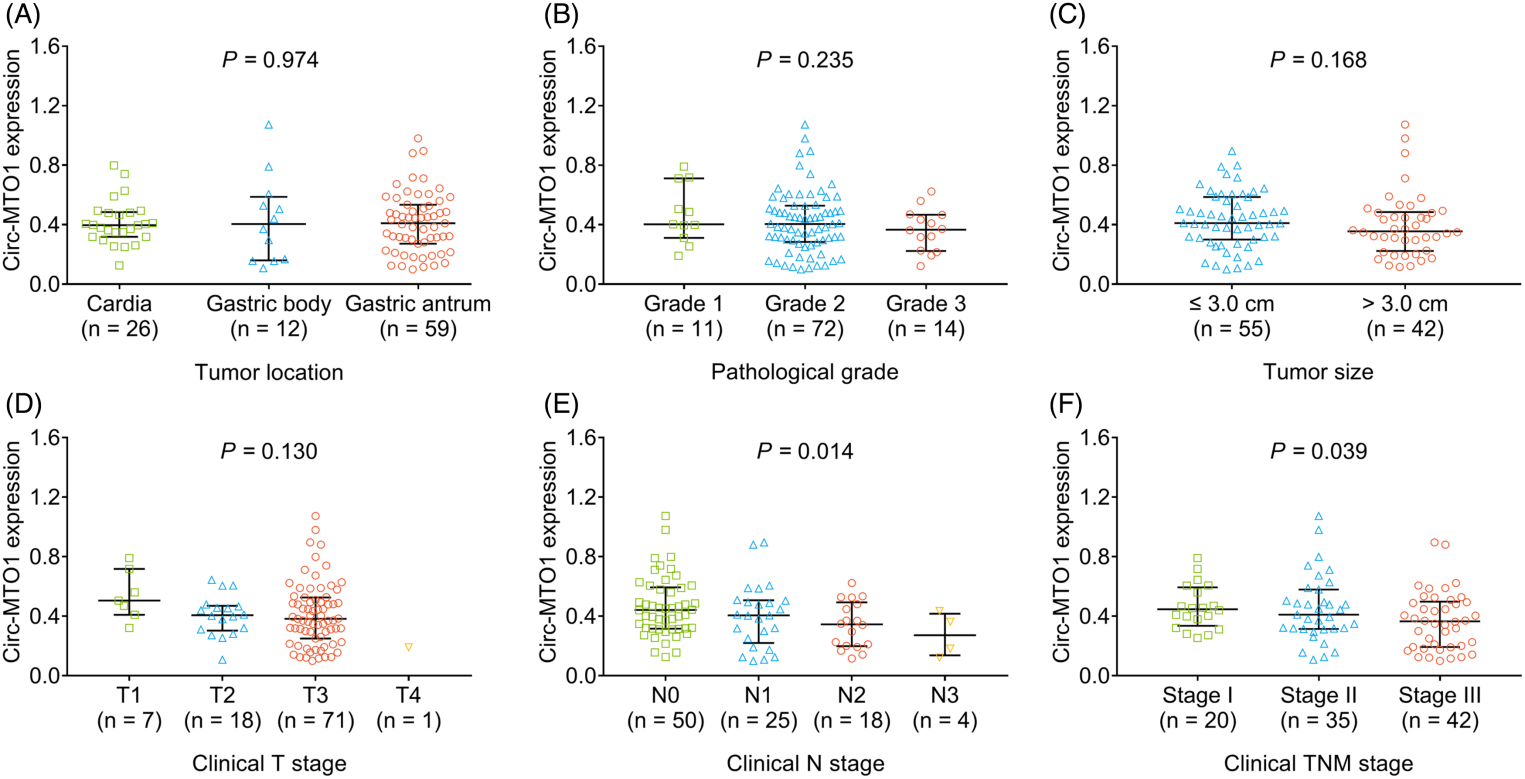
Association between tumor circ‐MTO1 and clinical characteristics. The association of tumor circ‐MTO1 expression with tumor location (A), pathological grade (B), tumor size (C), clinical T stage (D), clinical N stage (E), and clinical TNM stage (F) in gastric cancer patients. Circ‐MTO1, circular RNA mitochondrial translation optimization 1; T, tumor; N, lymph node; TNM, tumor‐node‐metastasis

Tumor circ‐MTO1 expression was negatively correlated with clinical N stage (*p *= 0.014) and clinical TNM stage (*p *= 0.039) (Figure 2E‐2F). However, no correlation of tumor circ‐MTO1 with tumor location, pathological grade, tumor size, or clinical T stage was observed (all *p *> 0.05) (Figure 2A‐2D). Besides, no correlation of tumor circ‐MTO1 with age, gender, or other clinical characteristics apart from tumor features was observed in gastric cancer patients (all *p *> 0.05; Table 2).

**TABLE 2:** Correlation of circ‐MTO1 with characteristics apart from tumor features

Items	Circ‐MTO1 expression Median (IQR)	*P* value
Age		
<60 years	0.384 (0.221–0.480)	0.293
≥60 years	0.429 (0.297–0.574)	
Gender		
Female	0.407 (0.295–0.532)	0.865
Male	0.395 (0.238–0.502)	
Current smoke		
No	0.383 (0.292–0.497)	0.541
Yes	0.448 (0.261–0.589)	
Current drink		
No	0.375 (0.256–0.493)	0.245
Yes	0.406 (0.318–0.544)	
Hypertension		
No	0.407 (0.297–0.502)	0.520
Yes	0.384 (0.185–0.527)	
Hyperlipidemia		
No	0.406 (0.254–0.509)	0.754
Yes	0.387 (0.308–0.527)	
DM		
No	0.398 (0.295–0.509)	0.935
Yes	0.424 (0.180–0.568)	
Helicobacter pylori infection		
Negative	0.405 (0.297–0.522)	0.855
Positive	0.384 (0.233–0.514)	
Adjuvant chemotherapy		
No	0.410 (0.318–0.560)	0.338
Yes	0.390 (0.223–0.513)	
Adjuvant radiotherapy		
No	0.405 (0.303–0.528)	0.113
Yes	0.226 (0.187–0.464)	

Abbreviations: Circ‐MTO1, circular RNA mitochondrial tRNA translation optimization 1; DM, diabetes mellitus; IQR, interquartile range.

### 3.4 Correlation of tumor circ‐MTO1 with accumulating DFS and OS

**FIGURE 3:**
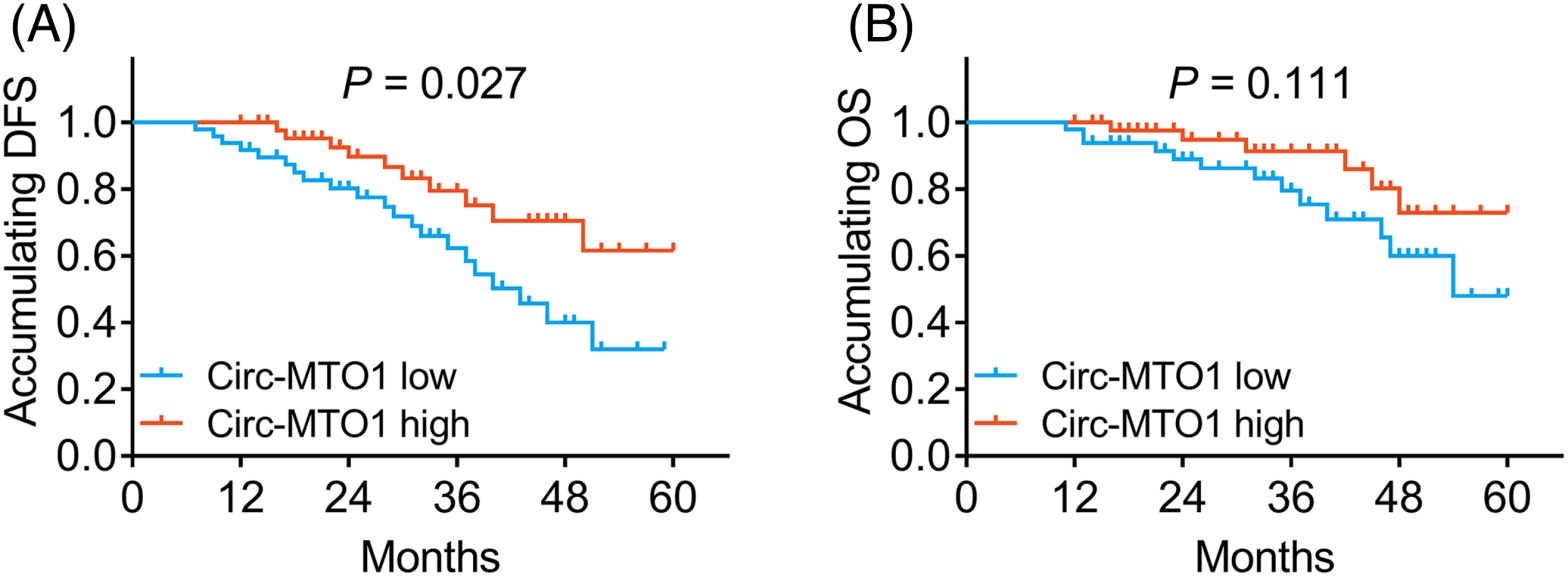
Association between tumor circ‐MTO1 and prognosis. The association of tumor circ‐MTO1 expression with accumulating DFS (A) and OS (B) in gastric cancer patients. Circ‐MTO1, circular RNA mitochondrial translation optimization 1; DFS, disease‐free survival; OS, overall survival

According to the median of circ‐MTO1 expression in gastric tumor tissues (Figure 1), tumor circ‐MTO1 expression was divided by high (over 0.403) and low (below 0.403) expressions. In gastric cancer patients, tumor circ‐MTO1 high expression was associated with better accumulating DFS (*p *= 0.027) (Figure 3A). In terms of accumulating OS, no correlation of tumor circ‐MTO1 with OS was found (*p *> 0.05) (Figure 3B).

### 3.5 Analysis of independent factors relating to DFS and OS

**FIGURE 4:**
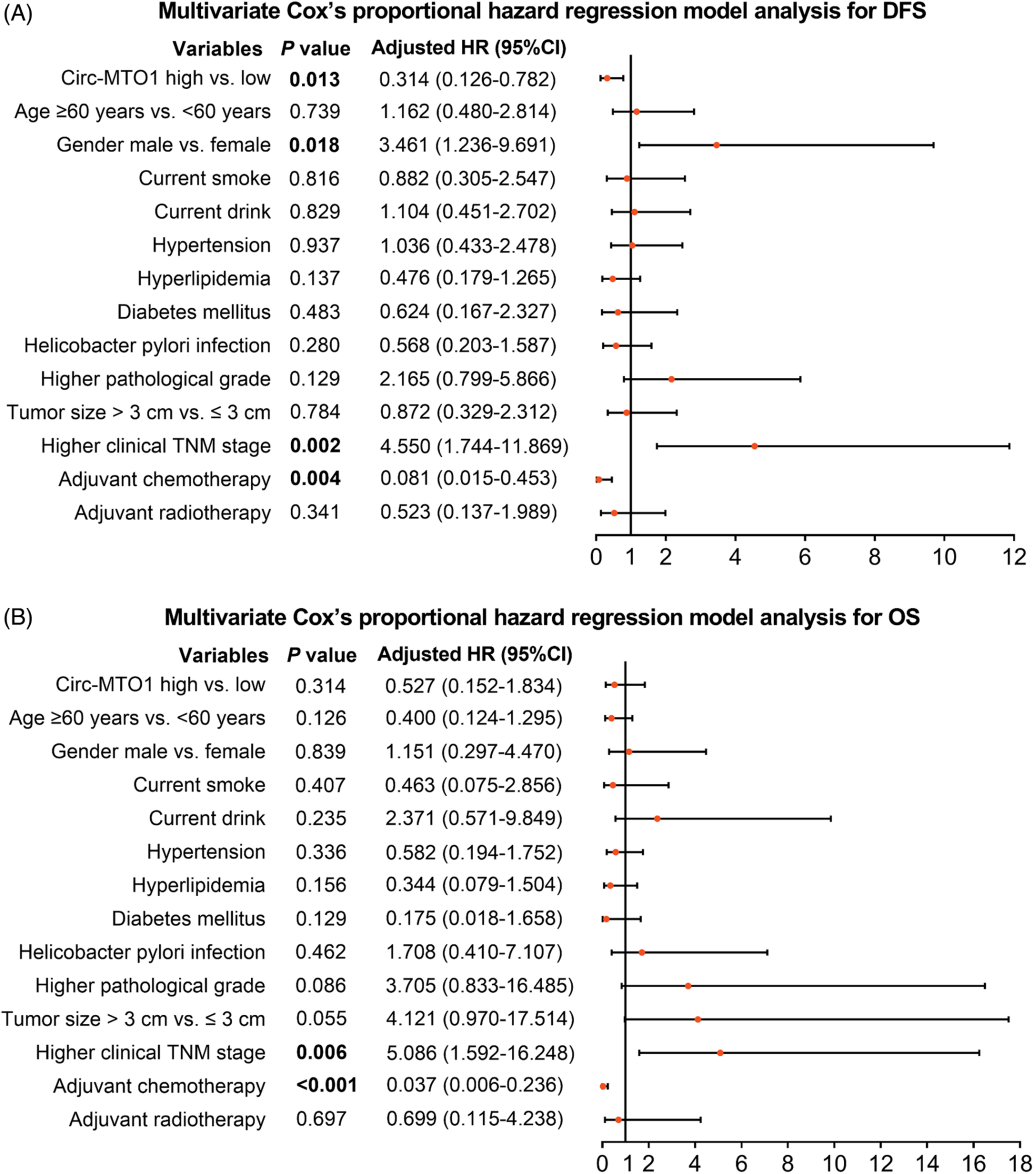
Multivariate regression analysis for DFS and OS. Factors affecting DFS (A) and OS (B) analyzed by multivariate Cox's proportional hazard regression model. DFS, disease‐free survival; OS, overall survival; HR, hazard ratio; CI, confidential interval; Circ‐MTO1, circular RNA mitochondrial translation optimization 1; TNM, tumor‐node‐metastasis

From multivariate Cox's proportional hazard regression model analyses for DFS (Figure 4A), high expression of tumor circ‐MTO1 (*p *= 0.013, adjusted hazard ratio (HR) (95% confidential interval (CI)): 0.314 (0.126–0.782)), and adjuvant chemotherapy (*p *= 0.004, adjusted HR (95%CI): 0.081 (0.015–0.453)) were independent factors for better DFS, while male (*p*=0.018, adjusted HR (95%CI): 3.461 (1.236–9.691)) and higher clinical TNM stage (*p*=0.002, adjusted HR (95%CI): 4.550 (1.744–11.869)) were independent factors for worse DFS. As to OS, adjuvant chemotherapy (*p*<0.001, adjusted HR (95%CI): 0.037 (0.006–0.236)) was an independent factor for better OS, while higher clinical TNM stage (*p*=0.006, adjusted HR (95%CI): 5.086 (1.592–16.248)) was an independent factor for worse OS (Figure 4B).

### 3.6 The effect of circ‐MTO1 on oxaliplatin sensitivity in gastric cancer cell lines

**FIGURE 5:**
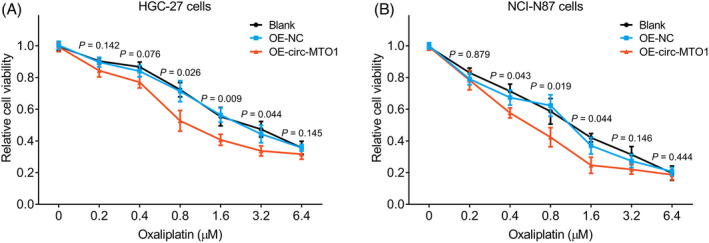
The influence of circ‐MTO1 on the sensitivity to oxaliplatin. The relative cell viability of OE‐circ‐MTO1, OE‐NC, and blank under oxaliplatin treatment in HGC‐27 (A) and NCI‐N87 (B) cell lines. Circ‐MTO1, circular RNA mitochondrial translation optimization 1

Since circ‐MTO1 independently correlated with PFS, we further explored the effect of circ‐MTO1 on oxaliplatin sensitivity in gastric cancer cell lines; then, plasmid transfection was conducted in HGC‐27 and NCI‐N87 cells followed by the oxaliplatin treatment. In terms of HGC‐27 cells, the relative cell viability of OE‐circ‐MTO1 group was lower than that of OE‐NC group at oxaliplatin concentrations of 0.8 μM, 1.6 μM, and 3.2 μM (all *p*<0.05) (Figure 5A). Besides, the same trend was observed in NCI‐N87 cells at oxaliplatin concentrations of 0.4 μM, 0.8 μM, and 1.6 μM (all *p*<0.05) (Figure 5B).

## 4 DISCUSSION

The main findings were listed as follows: 1) Circ‐MTO1 was down‐regulated in tumor tissue compared with adjacent tissue of gastric cancer patients. 2) Tumor circ‐MTO1 was correlated with less lymph node metastasis and lower clinical TNM stage in gastric cancer patients. 3). Circ‐MTO1 high expression was independently correlated with prolonged DFS, but was not correlated with OS in gastric cancer patients. 4) Circ‐MTO1 enhanced the sensitivity to oxaliplatin in gastric cancer cell lines.

CircRNAs, widely presenting in transcriptomes, play vital roles in the regulation of various biological activities.^25^ Circ‐MTO1 is one of the commonly studied circRNAs, which acts as a cancer‐suppressor gene involved in a variety of cancers.^19^, ^20^, ^21^, ^22^ circ‐MTO1 suppresses the growth and invasion of gastric, colorectal, and prostate cancer cells.^19^, ^20^, ^21^, ^22^ Moreover, it is insufficiently expressed in hepatocellular tumor tissues.^21^ In this study, circ‐MTO1 was down‐regulated in gastric tumor tissue compared with adjacent tissue, which could be explained by that overexpression of circ‐MTO1 inhibited the proliferation of gastric cancer cells via multiple mechanisms such as regulating miR‐199a‐3p/PAWR axis, miR‐3200‐5p/PEBP1 axis, miR‐9/p21 axis, and Wnt/β‐catenin signaling pathway.^19^, ^21^, ^22^, ^23^ The viability of gastric cancer cells was decreased, leading to its down‐regulation in gastric tumor tissue compared with adjacent tissues.

Circ‐MTO1 is clinically correlated with tumor characteristics.^19^, ^20^ For example, a study shows that insufficient circ‐MTO1 expression is associated with advanced lymph node metastasis and TNM stage in colorectal cancer patients.^19^ Another study exhibits that circ‐MTO1 is associated with decreased pathological T stage and N stage in patients with prostate cancer.^20^ Partly in line with these reports, our study presented that tumor circ‐MTO1 was correlated with less severe lymph node metastasis and clinical TNM stage in gastric cancer patients. The possible reasons might be that (1) circ‐MTO1 suppressed the invasion and migration of gastric cancer cells by up‐regulating PAWR via sponging miR‐199a‐3p,^22^ leading to the less severe lymph node metastasis in gastric cancer patients; (2) circ‐MTO1 also attenuated the proliferation and invasion, as well as promoted the apoptosis of gastric cancer cells via regulating the tumor necrosis factor receptor‐associated factor 4/Eg5 axis,^26^ resulting in lower TNM stage in gastric cancer patients.

As for prognosis of cancer patients, circ‐MTO1 is associated with their better survivals.^19^, ^20^, ^21^ For instance, circ‐MTO1 expression is associated with longer OS in patients with colorectal cancer and hepatocellular carcinoma.^19^, ^21^ Furthermore, circ‐MTO1 is independently correlated with favorable DFS and OS in prostate cancer patients.^20^ The current study also displayed that tumor circ‐MTO1 was correlated with prolonged DFS and was an independent factor affecting DFS in gastric cancer patients. The explanations might be as follows: (1) Tumor circ‐MTO1 was associated with less severe tumor metastasis and TNM stage, thus prolonging DFS in gastric cancer patients. (2) Circ‐MTO1 enhanced the sensitivity of chemotherapy which could bring a favorable treatment efficacy, leading to a subsequently longer DFS. This could be supported by the result that circ‐MTO1 increased the sensitivity of gastric cancer cell lines to oxaliplatin in this study. Furthermore, no correlation of circ‐MTO1 with OS in gastric cancer patients was observed in this study. This was attributed by that the number of death events was too small, leading to the low statistical power. Further study with long follow‐up duration was supposed to be conducted in the future.

It could not be ignored that there were a few limitations in this study. Firstly, the sample size was relatively small, which might lead to poor statistical power. Further study with a larger sample size was supposed to be performed. Secondly, specific mechanism about how circ‐MTO1 regulated gastric cancer progression was still unclear and was required to be extensively explored in the future. Lastly, since our study was a cohort study, selection bias and confounding factors such as diversified resection treatments and tumor status might exist.

Conclusively, circ‐MTO1 correlates with less lymph node metastasis, prolonged DFS, and increased chemotherapy sensitivity in gastric cancer. This study provides evidences for selecting optimized treatment strategies and improving prognosis for gastric cancer patients.

## CONFLICT OF INTEREST

No potential conflict of interest was reported by the authors.

## Data Availability

The data that support the findings of this study are available in this article.
